# Revisiting Brucellosis in Small Ruminants of Western Border Areas in Pakistan

**DOI:** 10.3390/pathogens9110929

**Published:** 2020-11-10

**Authors:** Tariq Jamil, Khushal Khan Kasi, Falk Melzer, Muhammad Saqib, Qudrat Ullah, Muhammad Roidar Khan, Maryam Dadar, Muhammad Haleem Tayyab, Stefan Schwarz, Heinrich Neubauer

**Affiliations:** 1 Institute of Bacterial Infections and Zoonoses, Friedrich-Loeffler-Institut, 07743 Jena, Germany; falk.melzer@fli.de (F.M.); heinrich.neubauer@fli.de (H.N.); 2 Institute of Microbiology and Epizootics, Department of Veterinary Medicine, Freie Universität, 14163 Berlin, Germany; stefan.schwarz@fu-berlin.de; 3 Institute of Epidemiology, Friedrich-Loeffler-Institut, 17493 Greifswald-Insel Riems, Germany; kkasi444@yahoo.com; 4 Disease Investigation Laboratory, Livestock and Dairy Development Department, Government of Baluchistan, Quetta 87300, Pakistan; 5 Department of Clinical Medicine and Surgery, Faculty of Veterinary Science, University of Agriculture, Faisalabad 38000, Pakistan; drmhkhan381@gmail.com; 6 Department of Theriogenology, Faculty of Veterinary Science, University of Agriculture, Faisalabad 38000, Pakistan; qudratmahsud@gmail.com (Q.U.); dr.roidadvet114@gmail.com (M.R.K.); 7 Department of Brucellosis, Razi Vaccine and Serum Research Institute, Agricultural Research, Education and Extension Organization (AREEO), Karaj 31975/148, Iran; dadar.m77@gmail.com

**Keywords:** Brucellosis, zoonosis, Baluchistan, KPK, Pakistan

## Abstract

Brucellosis, globally known bacterial zoonosis, is endemic to Pakistan. *B. abortus* in bovines, *B. melitensis* in small ruminants and *B. canis* in dogs mainly cause this disease. A total of 1821 sera (1196 from sheep and 625 from goats) from animal herds near the Pakistan–Afghanistan border were collected. In parallel testing of sera for anti-*Brucella* antibodies (*B. abortus* and *B. melitensis*) was carried out by RBPT and indirect ELISA. The presence of *Brucella* DNA in sera was tested by real-time PCR. The overall percentage of seropositive samples was 0.99 (18/1821) by both tests. All positive samples originated from Baluchistan territory which translated into 1.76% (18/1021). None of the positive sera had signals for *Brucella* DNA and none of sera from goats carried detectable antibodies. Both tests showed an almost perfect agreement with Kappa statistics. The flock size was found to be associated with the presence of anti-*Brucella* antibodies. The samples of Khyber Pakhtunkhwa (KPK) tested negative in both serological tests and hence were not processed for real-time PCR. The present study shows the presence of anti-*Brucella* antibodies in sheep in the Baluchistan region of Pakistan. Diagnostic services need to be improved and test and slaughter policies might be implemented for eradication of *Brucella* infection in these areas. Awareness about the infection is needed at the farmer’s level. Isolation and molecular biology of the isolates could help with understanding the prevailing etiology in a better way.

## 1. Introduction

Brucellosis is an important worldwide bacterial zoonosis caused by *Brucella* (*B.*) belonging to the family *Brucellaceae* and order *Rhizobiales*. These are Gram-negative, non-motile, intracellular living and non-spore forming coccobacilli. Depending upon the host preference, *B. abortus* infects bovines and wild animals, *B. melitensis* small ruminants, *B. canis* dogs, *B. suis* pigs and *B. ovis* rams [1], however spillover is possible [2,3]. It is characterized by late term abortion, retention of fetal membranes, orchitis and epididymitis resulting in infertility. Infected animals remain carriers and act as source of transmission to healthy animals [4]. Humans contract brucellosis via ingestion of contaminated raw milk or by accidental exposure to infected animals [5,6,7]. Economically, it is important in terms of abortion, screening and culling of animals and milk loss especially in developing countries. Diagnosis mainly depends on serology e.g., rose bengal plate test (RBPT), enzyme linked immunosorbent assay (ELISA), complement fixation test (CFT) and for bovine milk the milk ring test (MRT). Molecular biological tests are used for specific detection and differentiation of the brucellosis etiology. Isolation of the bacteria remains a gold standard but is hazardous for human health and requires advanced biosafety levels (i.e., level 3) and good training. Despite availability of newer and effective antimicrobials, the treatment of farm animals for brucellosis is generally forbidden as it poses significant public health risks [8,9,10].

Pakistan is an agriculture-based country where livestock plays an integral role in the national agriculture Gross Domestic Product (GDP). Bovines share a large proportion of the main livestock rearing areas whereas small ruminants play important roles in areas with harsh climate and scarcity of green fodder, such as Baluchistan and Khyber Pakhtunkhwa (KPK). Brucellosis is considered endemic in ruminants in Pakistan [11]. Previous studies have reported only serologic estimates of brucellosis in these areas. Our study was aimed to investigate *Brucella* spp. infection in sheep and goat populations living in neglected, remote, uncontrolled animals’ traffic areas bordering to Afghanistan using serological (e.g., RBPT and indirect ELISA) and molecular (e.g., real-time polymerase chain reaction) methods.

## 2. Materials and Methods

### 2.1. Study Area and Sampling Strategies

**Figure 1:**
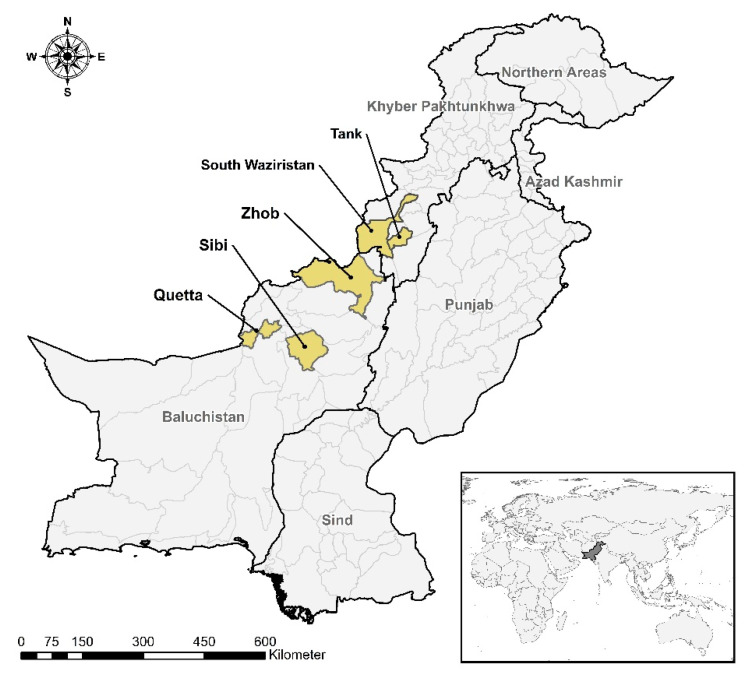
Areas sampled for brucellosis in Pakistan.

Baluchistan province is the largest province in the country by land area and is located in the southwest of Pakistan between 27°42′ N–65°42′ E (Figure 1). The total livestock population in Baluchistan is 28.08 million (19.66% of total population of the country) [12]. The climate is arid to semi-arid and the animals are often raised on pastures or natural flora. There is a seasonal migratory movement of humans and animal populations across the provincial and the Pakistan–Afghanistan borders [13,14]. For the sampling in Baluchistan, the sample size was calculated assuming an expected population prevalence of 50% with 95% confidence level and 10% desired precision [15]. A total of 106 flocks (53 livestock farms and 53 households) were identified, where up to 10 animals per farm/household were sampled randomly in each division, i.e., Quetta, Sibi and Zohb. Thus, a total of 1021 sera (596 sheep and 425 goats) were collected during June–July 2016.

Khyber Pakhtunkhwa (KPK) province is located to the northwest of Pakistan between 34°0′ N–71°19′ E (Figure 1). KPK bears a total livestock population of 21.62 million (15.14% of total population of the country) [12]. The climate varies from very hot (38–43 °C) in summer to moderately cold (2–4 °C) in winter. South Waziristan and Tank districts are located in the southwest of the province. The sample size was calculated assuming an expected prevalence of 50%, 95% confidence level and 5% desired precision [15]. This resulted in 384 samples, which were rounded up to 400 samples for each district. A total of 800 sera (600 sheep and 200 goats) were collected randomly.

None of the animals had a history of vaccination against brucellosis. All farms visited produced milk and meat for domestic consumption and rarely for commercial purposes.

### 2.2. Statement of Ethics 

In Baluchistan, blood samples were collected from animals of the farmers willing to participate in the study as per ethical and standard procedures defined by the Disease Investigation Laboratory, Livestock and Dairy Development Department, Government of Baluchistan, Quetta, Pakistan. In KPK, blood samples were collected from the animals with the help of local Veterinary officials as per standard operation procedures of Livestock and Dairy Development Department, Government of KPK, Pakistan and at the free will of the animal owners to participate in the study. No discrimination was done against apparently healthy or affected animals. No animals were killed/harmed during blood collection and restraining process. No follow up was done to the animals after the study. A written consent of the free will to participate in the study could not be possible as the majority of the animal owners was illiterate.

### 2.3. Serological Analysis

A total of 1821 sera (1196 sheep and 625 goats) were screened by RBPT (IDEXX, Montpellier, France). In tandem, both positive and negative control sera provided by the National Reference Laboratory (NRL) for *Brucella* spp. infections at Friedrich-Loeffler-Institut (FLI), Jena, Germany were tested following Office International des Epizooties (OIE) procedure [16]. The sera were tested in parallel by ID Screen^®^ (IDvet, Grabels, France) indirect ELISA for detection of livestock brucellosis (*B. abortus, B. melitensis* and *B. suis*) antibodies. The ELISA was performed and results calculated as per manufacturer’s recommendations.

### 2.4. Molecular Analyses

The samples were subjected to DNA extraction by High Pure Template Kit (Roche, Rotkreuz, Switzerland) and real-time PCR for detection and differentiation of *Brucella* spp. as described by [17] by using MX3000P qPCR machine (Agilent Technologies, Waldbronn, Germany). The primers and the target genes amplified are described in Supplementary Materials (Table S1). PCR conditions were as follows: decontamination at 50 °C for 2 min, 1 cycle, initial denaturation at 95 °C for 10 min, 1 cycle denaturation at 95 °C for 25 s and 1 cycle for annealing of the primers/elongation at 57 °C for 1 min. The last two cycles were repeated 50 times. All DNA extraction procedures were accompanied by *E. coli* controls whereas real-time PCR runs were accompanied by positive controls of *B. abortus* (ATCC No. 23448) and *B. melitensis* (ATCC No. 23456) reference strains from NRL for *Brucella* spp. infections at FLI, Jena, Germany. Although antigenically similar, *B. suis* control was not included in the runs as no *B. suis* has been reported in the country to date. RNAse/DNAse free water was used as no template negative control (NTC). The method was used and validated with camel sera [18]. Based on further in-house validation criteria, a cycle threshold (Ct) value of ≤38 was considered as positive in this study.

### 2.5. Statistical Analysis

Univariable analysis was performed by using Fisher’s exact test. Farm management related variables were considered as independent variables and the presence or absence of brucellosis-specific antibodies was considered as the dependent variable. Statistical tests were performed by Epi Info^TM^ 7 software (Centers for Disease Control and Prevention, Atlanta, GA, USA).

## 3. Results

The overall seropositivity in sheep and goats was 0.99% (18/1821) by both RBPT and indirect ELISA in both areas combined. All seropositive sera originated from sheep in Baluchistan. No seropositive samples were found from goats in Baluchistan and from both sheep and goats in KPK. All seropositive samples were from sheep and were from Zhob division in Baluchistan and were positive by both RBPT and indirect ELISA.

In Baluchistan, the overall seroprevalence was 1.76% (18/1021) by both by RBPT and indirect ELISA; however, all 18 seropositive samples originated from sheep. None of the sera contained *Brucella* DNA as confirmed by negative real-time PCR results. Flock-based seroprevalence was found to be 10.38% (11/106) in Baluchistan, with all seropositive flocks originating from the Zhob district. Among farm management related variables, flock size was found significantly (*p* < 0.05) associated with a seropositive status. Other variables e.g., presence of cattle, dogs and equines at the farm, quarantine measures, contact with other farm animals, farm hygiene and proper disposal of aborted materials were not found to be significantly (*p* > 0.05) associated (Table 1). Statistical analysis for goats was not possible as no seropositive samples were found.

In KPK, no sera were found seropositive in both sheep and goats, hence were not used for real-time PCR. In this regard, statistical analysis for animal- and farm-related variables was not possible. Hence, the related variables were analyzed descriptively, in particular to the practices which may be associated with brucellosis infection among livestock in this area.

In total, 1821 serum samples were tested by RBPT and iELISA. Out of these, 18 samples were found positive by RBPT and iELISA (Table 2). An inter-rater reliability analysis using the Kappa statistic was performed to determine agreement among two tests. Agreement between RBPT and iELISA results was found to be almost perfect (Kappa value = 1.000; 95% CI = 1.000–1.000; Standard Error = 0.00).

## 4. Discussion

Serology is an important method to detect the infection burden of *Brucella* infection in animals and to monitor the effectiveness of countermeasures. ELISA provides a sensitive and specific diagnosis and is widely used in monitoring of brucellosis. Conversely, RBPT is a cheaper, sensitive and readily available field screening test; however, it has lower diagnostic specificity [19]. Nevertheless, it remains the most adequate screening test to be used in the absence of a gold or validated standardized test and in resource-limited situations [16,20,21]. Hence, we used RBPT and indirect ELISA and compared the agreement of the two tests. Real-time PCR provides more specific and higher sensitivity diagnostic solutions than conventional PCR. Notwithstanding, diagnostic tests including serology and PCR may have their own limitations depending upon the time of sampling and the onset of infection in the animal/patient [22,23].

Previously, in Baluchistan, seroprevalences of 1.08% (2/185) in sheep and 0.55% (1/180) in goats were reported by RBPT in the district Quetta, whereas no seropositive animals were found in district Pishin [24]. Another study reported 2.66% (4/150) seroprevalence in sheep and 2.00% (3/150) in goats by RBPT in district Kech [25]. Other studies have reported a seroprevalence between 3.5–4.1% determined by RBPT in sheep and goats in Baluchistan and KPK [26,27,28]. The low prevalence in our study might be associated with the lower animal density and limited exposure of the animals compared to the areas where more contact would be possible e.g., animal sale/purchase market, slaughterhouses or animal exhibition areas. [29,30].

In KPK, a seropositivity report of 10% (10/100) in sheep by RBPT and SAT does exist in Kohat [31]. *Brucella* DNA was also found in 11% of bovines and 18% (9/50) of occupationally exposed humans in the district Bannu and in 14% (14/100) of buffaloes in the district Sawat by PCR [32,33]. A recent study indicated 15% and 6% SPAT seropositive cattle and humans, respectively, and confirmed the presence of both *B. abortus* and *B. melitensis* DNA by PCR [34]. Previously, *B. melitensis* has been isolated from caprine in KPK [35]. Even *Brucella* has been isolated from seronegative animals with previous history of abortion [29,30]. In this scenario, the largely negative results in our study cannot be related to the absence of infection in this area.

The age of the animals was not found significantly associated (*p* > 0.05) with the seroprevalence in our study like in previous studies. Previously, it has been found to be associated significantly [26,27,36]. In KPK, however, the statistical significance could not be determined as all the samples showed negative results.

A statistical association with the sex of the animal could not be determined as only males (*n* = 527) showed positive results. The samples from female animals (*n* = 105) showed negative results in serology in Baluchistan. It might be attributed to more male animals in the herds because of husbandry practices, although the real reason remains undetermined. This might have influenced our findings as reported previously [37,38]. However, statistically non-significant results are also reported [28,31,36,39,40,41].

History of reproductive disorders could not be determined because only male seropositive sera were found. Statistically significant results, however, do exist in association with the infection in small ruminants [14,41]. However, further studies with larger sample sizes are needed. Among KPK samples, the lambing/kidding history showed that among sheep, 14.95% suffered from stillbirths and 4.32% had premature birth. In the last 6 months before sampling, 47.5% of sheep and 29.5% of goats had a history of abortion.

Among farm management related variables, overall flock size was found to be statistically significant (*p* < 0.05) when associated with seroprevalence. More seropositive samples were found in smaller herds (animals ≤ 20) than in larger herds (21–100 and > 100). This contrasts with previous studies that found statistically significant associations in larger herds [42,43]. However, a non-significant association of the flock size has also been reported and might be because of the differences in husbandry practices based on each sampling tier [44]. Other factors e.g., body condition, contact to other animals, quarantine practices, farm hygiene and sharing of the grazing area were not found statistically significant (*p* > 0.05) with the seroprevalence of brucellosis.

The median time for seroconversion ranges between 2–4 weeks for both *B. abortus* and *B. melitensis* which may influence the diagnostic results [45]. The lack of awareness, over-the-counter availability of antimicrobials/antipyretics, and animal movements across administrative borders combined with poor animal recording systems might be negatively affecting proper detection and reporting of *Brucella* infections, which could be a pitfall for zoonosis control [14,46,47,48]. Pakistan has been one of the highest dairy milk producing countries in the world and almost 97% of the milk produced is purchased as raw/unpasteurized [49,50]. Population explosions and ever-increasing demand for milk might result in an increasingly intensive livestock farming trend in the future. The same could be expected for sheep and goat milk. Human brucellosis has been reported (3.6–21.7%) in the country depending upon the exposure level, region and type of the diagnostic tool used [32,34,35,51,52,53,54]. Thus, brucellosis in animals will remain a continuous public health significant threat-if not controlled. Although treatment of brucellosis in animals may become an alternative to test-and-slaughter policy, no 100% effective treatment regimens have been developed to date [55,56]. Understanding transmission, genetic relatedness and variation at molecular level of the local isolates might help to take countermeasures swiftly.

## 5. Conclusions

The low-level seroprevalence of anti-*Brucella* antibodies reflects the persistent endemicity of the infection in small ruminants in the study area. Based on this finding, test and slaughter policies in small ruminants may be advocated [57]. All animals needed to be marked for the sake of record keeping. Milk marketing business needs to be improved for traceability of products. Pasteurization may be helpful. A better understanding and coordination of actions is needed between farmers and concerned veterinary and medical public health authorities to develop infection prevention and control policies in case of accidental outbreaks. To the best of our knowledge, this is the first study applying a statistics-based sampling regime for brucellosis in small ruminant populations in the western areas of Pakistan. Public awareness programs and strict biosecurity and record keeping may help prevent infection transmission. Appropriate risk assessment and funding for such programs is necessary. Suspected and clinically infected animals should be the focus for future studies where brucellosis is frequently reported as their history could help in identifying factors and drawing conclusions about the countermeasures. Regular screening of herds is necessary especially before bringing new animals into an existing herd. Improvement of diagnostic systems and facilities for implementation of culture examination for samples from humans and animals remains overdue.

## Figures and Tables

**Table 1:** Univariable analysis of farm management related variables in Baluchistan (*n* = 106).

Variable	OR	95% CI	df	* *p*-Value
Flock size				<0.001
Small (≤20) (*n* = 41)	0.88	0.19–3.95	1	1.00
Medium (21–100) (*n* = 14)	Ref			
Large (>100) (*n* = 51)				
Presence of cattle				
Yes (*n* = 77)	─	─	1	0.282
No (*n* = 29)				
Presence of Dogs				
Yes (*n* = 97)	─	─	1	0.593
No (*n* = 9)				
Presence of equine				
Yes (*n* = 22)	─	─	1	0.115
No (*n* = 84)				
Quarantine of newly introduced animals				
Yes (*n* = 7)	─	─	1	1.00
No (*n* = 99)				
Farm animal contact with other farm animals				
Yes (*n* = 82)	─	─	1	0.265
No (*n* = 24)				
Grazing area used by animals of other farms				
Yes (*n* = 83)	0.28	0.07–1.02	1	0.058
No (*n* = 23)	Ref			
Farm hygiene				
Satisfactory (*n* = 39)	3.44	0.94–12.64	1	0.094
Poor (*n* = 67)	Ref			
Proper disposal of aborted fetus				
Yes (*n* = 14)	─	─	1	0.637
No (*n* = 92)				

OR: odds ratio; CI: confidence interval; df: degree of freedom; *p*-value: Fisher’s exact test; Ref: reference; *: *p*-value < 0.05 considered as significant.

**Table 2:** Comparison of results of RBPT (rose bengal plate test) and iELISA (indirect enzyme linked immunosorbent assay) tests used to detect anti-*Brucella* antibodies in small ruminants.

RBPT	iELISA		Total
	Negative	Positive	
Negative	1803	18	1803
Positive	0	18	18
Total	1803	18	1821

## Supplementary Materials

The following are available online at https://www.mdpi.com/2076-0817/9/11/929/s1, Table S1: Primer and probes sequence for real-time PCR.

## References

[1] Diaz Aparicio E. (2013). Epidemiology of brucellosis in domestic animals caused by *Brucella melitensis, Brucella suis* and *Brucella abortus*. Rev. Sci. Tech..

[2] Jamil T., Melzer F., Khan I., Iqbal M., Saqib M., Hammad Hussain M., Schwarz S., Neubauer H. (2019). Serological and molecular investigation of *Brucella* species in dogs in Pakistan. Pathogens.

[3] Saleem M.Z., Akhtar R., Aslam A., Rashid M.I., Chaudhry Z.I., Manzoor M.A., Shah B.A., Ahmed R., Yasin M. (2019). Evidence of *Brucella abortus* in non-preferred caprine and ovine hosts by real-time PCR assay. Pak. J. Zool..

[4] Pérez-Sancho M., García-Seco T., Domínguez L., Álvarez J. (2015). Control of animal brucellosis, The most effective tool to prevent human brucellosis. Updates Brucell..

[5] Dadar M., Fakhri Y., Shahali Y., Mousavi Khaneghah A. (2020). Contamination of milk and dairy products by *Brucella* species: A global systematic review and meta-analysis. Food Res. Int..

[6] Ali S., Ali Q., Neubauer H., Melzer F., Elschner M., Khan I., Abatih E., Ullah N., Irfan M., Akhter S. (2013). Seroprevalence and risk factors associated with brucellosis as a professional hazard in Pakistan. J. Foodborne Pathog. Dis..

[7] Traxler R.M., Guerra M.A., Morrow M.G., Haupt T., Morrison J., Saah J.R., Smith C.G., Williams C., Fleischauer A.T., Lee P.A. (2013). Review of brucellosis cases from laboratory exposures in the United States in 2008 to 2011 and improved strategies for disease prevention. J. Clin. Microbiol..

[8] Aragón-Aranda B., de Miguel M.J., Martínez-Gómez E., Zúñiga-Ripa A., Salvador-Bescós M., Moriyón I., Iriarte M., Muñoz P.M., Conde-Álvarez R. (2019). Rev1 wbdR tagged vaccines against *Brucella ovis*. Vet. Res..

[9] Jamil T., Melzer F., Njeru J., El-Adawy H., Neubauer H., Wareth G. (2017). *Brucella abortus*: Current research and future trends. Curr. Clin. Microbiol. Rep..

[10] Al-Sherida Y., El-Gohary A.H., Mohamed A., El-Diasty M., Wareth G., Neubauer H., Abdelkhalek A. (2020). Sheep Brucellosis in Kuwait: A Large-Scale serosurvey, identification of *Brucella* species and zoonotic significance. Vet. Sci..

[11] Farooq U., Fatima Z., Afzal M., Anwar Z., Jahangir M. (2011). Sero-prevalence of brucellosis in bovines at farms under different management conditions. Br. J. Dairy Sci..

[12] (2006). Livestock Population.

[13] Fazl-e-Haider S. (2008). Issues in Balochistan’s livestock development. DAWN.

[14] Khan A.Q., Haleem S.K., Shafiq M., Khan N.A., Rahman S.U. (2017). Seropositivity of brucellosis in human and livestock in tribal-Kurram agency of Pakistan indicates cross circulation. Thai J. Vet. Med..

[15] Cannon R., Roe R. (1982). Livestock Disease Surveys. A Field Manual for Veterinarians.

[16] OIE (2018). Brucellosis (*Brucella abortus, B. melitensis* and *B. suis*) (infection with *B. abortus, B. melitensis* and *B. suis*). Manual of Diagnostic Tests and Vaccines for Terrestrial Animals.

[17] Probert W.S., Schrader K.N., Khuong N.Y., Bystrom S.L., Graves M.H. (2004). Real-time multiplex PCR assay for detection of *Brucella* spp., *B. abortus*, and *B. melitensis*. J. Clin. Microbiol..

[18] Gwida M.M., El-Gohary A.H., Melzer F., Tomaso H., Rosler U., Wernery U., Wernery R., Elschner M.C., Khan I., Eickhoff M. (2011). Comparison of diagnostic tests for the detection of *Brucella* spp. in camel sera. BMC Res. Notes.

[19] Nielsen K. (2002). Diagnosis of brucellosis by serology. Vet. Microbiol..

[20] Ducrotoy M.J., Munoz P.M., Conde-Alvarez R., Blasco J.M., Moriyon I. (2018). A systematic review of current immunological tests for the diagnosis of cattle brucellosis. Prev. Vet. Med..

[21] Gusi A.M., Bertu W.J., de Miguel M.J., Dieste-Perez L., Smits H.L., Ocholi R.A., Blasco J.M., Moriyon I., Munoz P.M. (2019). Comparative performance of lateral flow immunochromatography, iELISA and Rose Bengal tests for the diagnosis of cattle, sheep, goat and swine brucellosis. PLoS Negl. Trop. Dis..

[22] Navarro E., Casao M.A., Solera J. (2004). Diagnosis of human brucellosis using PCR. Expert Rev. Mol. Diagn..

[23] Dal T., Kara S.S., Cikman A., Balkan C.E., Acikgoz Z.C., Zeybek H., Uslu H., Durmaz R. (2019). Comparison of multiplex real-time polymerase chain reaction with serological tests and culture for diagnosing human brucellosis. J. Infect. Public Health.

[24] Batool B.T., Tareen A., Ahmed S.S., Ejaz H., Kakar M.A., Rehman S.A., Saeed M., Ahmad Z., Tariq M.M., Awan M.A. (2017). Prevalence of zoonotic tuberculosis and brucellosis in animals of Quetta and Pishin districts, Balochistan. Pak. J. Zool..

[25] Shafee M., Ahmed N., Razzaq A., ur Rehman F., Yakoob M. (2016). Seroprevalence of brucellosis in small ruminants in Turbat (Kech), Balochistan. Lasbela Univ. J. Sci. Technol..

[26] Achakzai M. (2008). A Study on the Seroprevalence of Brucellosis in Caprine in Balochistan.

[27] Naeem K., Kamram J., Ullah A. Seroprevalence and risk factors associated with Crimean-Congo haemorrhagic fever and brucellosis in people and livestock in Baluchistan and Khyber Pakhtunkhwa Provinces, Pakistan. Proceedings of the South Asia Regional One Health Symposium.

[28] Ali S., Akbar A., Shafee M., Tahira B., Muhammed A., Ullah N. (2017). Sero-epidemiological study of brucellosis in small ruminants and associated human beings in district Quetta, Balochistan. Pure Appl. Biol..

[29] El-Diasty M., Wareth G., Melzer F., Mustafa S., Sprague L.D., Neubauer H. (2018). Isolation of *Brucella abortus* and *Brucella melitensis* from seronegative cows is a serious impediment in brucellosis control. Vet. Sci..

[30] Islam M.S., Islam M.A., Khatun M.M., Saha S., Basir M.S., Hasan M.M. (2018). Molecular detection of *Brucella* spp. from milk of seronegative cows from some selected area in Bangladesh. J. Pathog..

[31] Hussain M., Rind R., Adil M., Khan M., Farmanullah S.A., Waheed U., Salim M. (2014). Seroprevalence of brucellosis in sheep and humans in district Kohat, Pakistan. Adv. Anim. Vet. Sci..

[32] Khan A., Shafee M., Khan N., Rahman A., Rafiullah, Ali I., Khan I., Rahman S.U. (2018). Incidence of brucellosis in aborted animals and occupationally exposed veterinary professionals of Bannu, Khyber Pakhtunkhwa, Pakistan. Thai J. Vet. Med..

[33] Khan S.I., Muti-ur-Rehmana S.M., Khanc A., Khand A., Shakeeld M., Shah M.A., Naseerd Z. (2017). Patho-epidemiology of bovine brucellosis in Aza-kheli buffalo in Pakistan. Veterinaria.

[34] Khan M.Z., Usman T., Sadique U., Qureshi M.S., Hassan M.F., Shahid M., Khan A. (2017). Molecular characterization of *Brucella abortus* and *Brucella melitensis* in cattle and humans at the north west of Pakistan. Pak. Vet. J..

[35] Mahmood R., Ali T., Waheed U., Asif M., Khan Q.M. (2016). Application of serum based PCR and fluorescence polarization assay for diagnosis of brucellosis among people occupationally at risk to disease. Int. J. Agric. Biol..

[36] Ullah Q., Jamil T., Melzer F., Saqib M., Hussain M.H., Aslam M.A., Jamil H., Iqbal M.A., Tahir U., Ullah S. (2020). Epidemiology and Associated Risk Factors for Brucellosis in Small Ruminants Kept at Institutional Livestock Farms in Punjab, Pakistan. Front. Vet. Sci..

[37] Gul S.T., Khan A., Rizvi F., Hussain I. (2014). Sero-prevalence of brucellosis in food animals in the Punjab, Pakistan. Pak. Vet. J..

[38] Iqbal Z., Jamil H., Qureshi Z.I., Saqib M., Lodhi L.A., Waqas M.S., Safdar M. (2013). Seroprevalence of ovine brucellosis by modified Rose Bengal test and ELISA in Southern Punjab, Pakistan. Pak. Vet. J..

[39] Din A.M., Khan S.A., Ahmad I., Rind R., Hussain T., Shahid M., Ahmed S. (2013). A study on the seroprevalence of brucellosis in human and goat populations of district Bhimber, Azad Jammu and Kashmir. J. Anim. Plant Sci..

[40] Ghani M., Siraj M., Zeb A., Naeem M. (1995). Sero-epidemiological study of brucellosis among goats and sheep at Pshawar district. Asian-Australas. J. Anim. Sci..

[41] Arshad M., Munir M., Iqbal K., Abbas R., Rasool M., Khalil N. (2011). Sero-prevalence of brucellosis in goats from public and private livestock farms in Pakistan. Online J. Vet. Res..

[42] Addis S.A., Desalegn A.Y. (2018). Comparative sero-epidemiological study of brucellosis in sheep under smallholder farming and governmental breeding ranches of central and north east Ethiopia. J. Vet. Med..

[43] Coelho A.C., Díez J.G., Coelho A.M. (2015). Risk factors for *Brucella* spp. in domestic and wild animals. Updates on Brucellosis.

[44] Al-Majali A.M., Majok A.A., Amarin N.M., Al-Rawashdeh O.F. (2007). Prevalence of, and risk factors for, brucellosis in Awassi sheep in southern Jordan. Small Rumin. Res.

[45] Mikolon A.B., Gardner I.A., Hietala S.K., de Anda J.H., Chamizo Pestana E., Hennager S.G., Edmondson A.J. (1998). Evaluation of North American antibody detection tests for diagnosis of brucellosis in goats. J. Clin. Microbiol..

[46] Khan A., Khan S., Abbas S.A., Khan M. (2018). Health complications associated with self-medication. J. Phys. Fit. Treat. Sports.

[47] Khurshid A., Hassan M., Alam M.M., Aamir U.B., Rehman L., Sharif S., Shaukat S., Rana M.S., Angez M., Zaidi S.S.Z. (2015). CCHF virus variants in Pakistan and Afghanistan: Emerging diversity and epidemiology. J. Clin. Virol..

[48] Aziz M.M., Masood I., Yousaf M., Saleem H., Ye D., Fang Y. (2018). Pattern of medication selling and self-medication practices: A study from Punjab, Pakistan. PLoS ONE.

[49] Zia U.-E. (2007). Analysis of milk marketing chain-Pakistan. Ital. J. Anim. Sci..

[50] Zia U.-E., Mahmood T., Ali M. (2011). Dairy Development in Pakistan.

[51] Ali S., Nawaz Z., Akhtar A., Aslam R., Zahoor M.A., Ashraf M. (2018). Epidemiological investigation of human brucellosis in Pakistan. Jundishapur J. Microbiol..

[52] Ali S., Akhter S., Neubauer H., Scherag A., Kesselmeier M., Melzer F., Khan I., El-Adawy H., Azam A., Qadeer S. (2016). Brucellosis in pregnant women from Pakistan: An observational study. BMC Infect. Dis..

[53] Mukhtar F. (2010). Brucellosis in a high risk occupational group: Seroprevalence and analysis of risk factors. J. Pak. Med. Assoc..

[54] Ahmad H., Ali I., Ahmad T., Tufail M., Ahmad K., Murtaza B.N. (2017). Prevalence of brucellosis in human population of district Swat, Pakistan. Pak. J. Zool..

[55] Yousefi-Nooraie R., Mortaz-Hejri S., Mehrani M., Sadeghipour P. (2012). Antibiotics for treating human brucellosis. Cochrane Database Syst. Rev..

[56] Rasool H., Avais M., Rabbani M., Ahmed R., Muhammad K., Ali M. (2018). An innovative approach to treat brucellosis in Buffaloes. Biomed. Lett..

[57] Nawaz G., Malik M.N., Mushtaq M.H., Ahmad F.M., Shah A.A., Iqbal F., Ali S.W., Fatima Z., Khan A. (2016). Surveillance of brucellosis in livestock in rural communities of Punjab. J. Agric. Res..

