# Supplementary material for: Revisiting Brucellosis in Small Ruminants of Western Border Areas in Pakistan
Source: Pathogens. 2020 Nov 10;9(11):929. doi: 10.3390/pathogens9110929 (PMC7697571; doi:10.3390/pathogens9110929)
Supplement: Supplementary file 1 [file pathogens-09-00929-s001.pdf]

Table S1: Primer and probes sequence for real-time PCR

Genes detected	Primers/Probes	Sequence (5'-3')	Reference
Brucella (bcsp31)	Forward Primer	GCTCGGTTGCCAATATCAATGC	[17]
	Reverse Primer	GGGTAAAGCGTCGCCAGAAG	
	Probe	FAM-AAATCTTCCACCTTGCCCTTGCCATCA-BHQ	
Brucella abortus (IS711)[1]	Forward Primer	GCGGCTTTTCTATCACGGTATTC	
	Reverse Primer	CATGCGCTATGATCTGGTTACG	
	Probe	HEX-CGCTCATGCTCGCCAGACTTCAATG-BHQ	
Brucella melitensis (IS711)[2]	Forward Primer	AACAAGCGGCACCCCTAAAA	
	Reverse Primer	CATGCGCTATGATCTGGTTACG	
	Probe	Cy5-CAGGAGTGTTTCGGCTCAGAATAATCCACA-BHQ	

[1]*alkB Brucella abortus biotype 1 (strain 544/*ATCC 23448)*, [2]BMEI1162 *Brucella melitensis biotype 1 (strain 16M/*ATCC 23456), *American Type Culture Collection*
